# Low-dose radiotherapy for COVID-19 pneumonia treatment: case report, procedure, and literature review

**DOI:** 10.1007/s00066-020-01675-z

**Published:** 2020-08-20

**Authors:** Ruben Del Castillo, David Martinez, Gustavo J. Sarria, Luis Pinillos, Bertha Garcia, Luis Castillo, Alicia Carhuactocto, Frank A. Giordano, Gustavo R. Sarria

**Affiliations:** 1 Department of Radiation Oncology, Clinica Delgado-AUNA, Lima, Peru; 2 Department of Critical Care, Clinica Delgado-AUNA, Lima, Peru; 3 Department of Radiation Oncology, University Hospital Bonn, Universitätsklinikum Bonn, Venusberg Campus 1, Building 55, 53127 Bonn, Germany

**Keywords:** COVID-19, Low-dose radiotherapy, Viral pneumonia, Intensive care, Cytokine storm

## Abstract

**Background:**

The COVID-19 pandemic outbreak has set the emergency services in developing countries on major alert, as the installed response capacities are easily overwhelmed by the constantly increasing high demand. The deficit of intensive care unit beds and ventilators in countries like Peru is forcing practitioners to seek preventive or early interventional strategies to prevent saturating these chronically neglected facilities.

**Case presentation:**

A 64-year-old patient is reported after presenting with COVID-19 pneumonia and rapidly progressing to deteriorated ventilatory function. Compassionate treatment with a single 1‑Gy dose to the bilateral whole-lung volume was administered, with gradual daily improvement of ventilatory function and decrease in serum inflammatory markers and oxygen support needs, including intubation. No treatment-related toxicity developed. Procedures of transport, disinfection, and treatment planning and delivery are described.

**Conclusion:**

Whole-lung low-dose radiotherapy seems to be a promising approach for avoiding or delaying invasive respiratory support. Delivered low doses are far from meeting toxicity ranges. On-going prospective trials will elucidate the effectiveness of this approach.

## Introduction

The coronavirus disease 2019 (COVID-19) pandemic outbreak in late 2019 eventually imposed upon developing countries a major problem for public health systems, due to a lack of installed attention capacity [1]. Examples from European countries such as Spain or Italy, who saw their emergency and hospitalization systems rapidly overwhelmed despite a greater response capacity [2], raised major concern in Latin American countries. In Peru, a major population mobility restriction was introduced on March 15 [3], in order to alleviate the admission rates in a country where, according to official numbers, the overall availability of mechanical ventilators (MV) does not exceed 822 for a total of ~33 million inhabitants [3]. Despite these efforts and as of May 25, the contagion incidence curves seem to still be increasing, with an average of more than 4000 new cases daily since the first official case was reported on March 6 [3, 4].

Currently, in light of the lack of evidence for a specific or effective medical treatment, hospital- and especially intensive care unit (ICU)-based support management have been left as the only options for the expected 5% of advanced cases [5]. According to this and in addition to the previously described situational features, the fragile balance between patients’ admission and attention capacity should be approached from a preventive perspective.

The anti-inflammatory effect of low-dose radiotherapy (LD-RT) was tested for infectious respiratory conditions in former times; however, this practice fell into decline after the advent of medical treatments [6]. Pre-clinical and clinical data regarding toxicity and effectiveness of such treatments have been published, showing an acceptable safety profile [7].

Herein, we present the case of a patient treated at our institution, describe the followed biosecurity and disinfection protocol, and review the available evidence regarding this topic published to date.

## Case description

We here report on a 64-year-old male patient with a prior history of pulmonary tuberculosis successfully treated in 1975. Ten days prior to admission, he noted malaise, headache, and myalgia, followed by the onset of fever and dry cough 5 days prior to admission. After rapid worsening of symptoms and onset of dyspnea, he presented in the emergency department, where COVID-19 was confirmed by polymerase chain reaction (PCR). Baseline vital signs were: heart rate (HR) 93 bpm, respiratory rate (RR) 22 bpm, temperature (T°) 37.7 °C, blood pressure 133/93 mm Hg, oxygen saturation level (SpO_2_) 89%.

Oxygen support therapy was started with a non-rebreather mask at 10 L/min output (FiO_2_ 100%) Pa/FiO_2_ 193, with immediate SpO_2_ improvement to 99%. Chest CT scan revealed a bilateral multifocal ground-glass pattern, with a predominant central and subpleural component. Type I respiratory insufficiency secondary to the viral infection was diagnosed and multidrug treatment including ceftriaxone 2 g intravenous (iv) 24 h, hydroxychloroquine 400 mg per oral (po) twice daily (bid) first day, then 200 mg po bid for 10 days, azithromycin 500 mg po first dose, then 250 mg po per day for 10 days, and enoxaparin 60 mg subcutaneous (sc) per day was started. After 48 h, oxygen support therapy was deescalated to nasal cannula (NC) to 5 L/min, reaching 94% SpO_2_ and dyspnea pattern improvement. Fever persisted, with T° 39.3 at highest point. Daily laboratory and arterial blood gas (ABG) analyses from therapy installation are summarized in Tables 1 and 2.

**Table 1:** Longitudinal evolution of blood values pre- and post-RT

Timepoint	Management onset (3rd and 2nd pre-RT days)		1st pre-RT day	24 h post-RT	2nd day	3rd day	4th day	5th day	8th day
*Hospital area*	CIPA	CIPA	CIPA	ICU	ICU	ICU	ICU	ICU	CIPA
*Hemoglobin g/dL (13.5–17.5)*	13.6	NR	13.8	13.2	13.2	13.1	13.3	NR	14.4
*Leucocytes/mm*^*3*^* (4500–11,000)*	4240	NR	9190	5880	5730	5290	6010	NR	5430
Bands (0–400)	0	NR	0	0	0	0	0	NR	108
Lymphocytes (1300–3500)	920	NR	2417	1181.9	1048	820	1220	NR	1303
Segmented (1400–6600)	NR	NR	NR	NR	NR	NR	NR	NR	NR
*Platelets/mm*^*3*^* (150,000–475,000)*	141,000	NR	167,000	230,000	276,000	319,000	351,000	NR	390,000
*D‑Dimer mg/L (0.0–0.5)*	0.45	NR	0.46	NR	1.33	1.32	1.13	NR	0.55
*GPT U/L (0.0–41.0)*	61	NR	130	124	117	NR	NR	NR	70
*AST U/L (0.0–40.0)*	65	NR	150	99	75	NR	NR	NR	33
*CRP mg/dL (0.0–0.5)*	14.99	NR	26.37	12.5	4.25	1.86	0.82	NR	0.24
*Procalcitonin ng/mL (0.0–0.5)*	0.15	NR	0.39	NR	0.16	NR	NR	NR	NR
*Troponin T ng/dL (0.0–0.05)*	0.01	NR	0.009	NR	0.006	NR	0.006	NR	0.007
*LDH U/L (135.0–225.0)*	393	NR	526	510	429	354	347	NR	260
*Ferritin ng/mL (20.0–250.0)*	1817	NR	925	NR	2418	1231.6	1034	NR	871.2
*IL‑6* *pg/mL (0–7)*	107.1	NR	151.1	NR	1544	1117	NR	NR	243.3

*CRP* C-reactive protein, *NR* not registered, *CIPA* COVID in-patient area, *RT* Radiotherapy, *ICU* Intensive-care unit, *GPT* glutamate-pyruvate transaminase, *AST* aspartate aminotransferase, *LDH* Lactate dehydrogenase, *IL-6* Interleukin 6

**Table 2:** Arterial blood gas analyses (ABG) pre- and post-RT

	3rd pre-RT day		2nd pre-RT	1st pre-RT	24 h post-RT	2nd day	3rd day	4th day	5th day	8th day
Ventilatory device	Baseline	Non-rebreather mask	NR	HFNC	HFNC	HFNC	HFNC	HFNC	HFNC	Binasal cannula
*FiO*_*2*_* (%)*	21	100	NR	50	50	50	40	40	40	30
*ABG*										
pH	NR	7.43	NR	7.42	7.39	7.41	7.38	7.38	7.4	7.41
pCO_2_	NR	34.6	NR	38.4	41.1	40	44.8	44.9	42.5	45.3
pO_2_	NR	193.1	NR	51.8	73.5	85.5	88.8	90.5	85.3	80.8
HCO_3_	NR	22.3	NR	24.2	24.2	25.1	25.8	25.7	25.7	28.4
BE	NR	2.1	NR	0.3	0.8	0.5	0.7	0.5	0.9	3.8
SatO_2_%	89	93	NR	92.5	91.2	96.6	96.9	96.9	NR	NR
PaO_2_/FiO_2_	NR	193	NR	104	147	171	222	226	213	269
Lactate	NR	2	NR	3.5	2.2	2	2.2	1.9	NR	1.9

*HFNC* high-flow nasal cannula, *NR* not registered

During the second day of hospitalization, the dyspnea worsened further and was present at rest. Oxygen therapy with a non-rebreather mask was intensified to 15 L/min, reaching 85% SpO_2_ and 246 Pa/FiO_2_. Empiric treatment with tocilizumab 600 mg IV was started and the antibiotic was switched to ceftazidime 2 g iv bid. Due to further deterioration and a high likelihood of fatality of the clinical infection, whole-lung RT was offered as compassionate treatment [8].

The third day continued with the prior installed management. Clinical and ABG analysis showed RR 25/min, SpO_2_ 91.2%, PO_2_ 73.5 mm Hg, FiO_2_ 0.5, and Pa/FiO_2_ 147. The following protocol was followed regarding biosecurity measures, treatment planning, and delivery: all equipment was precovered with disposable plastic barriers. The patient was transported from the hospitalization unit with a fully isolated transport bed, through a designated transit area at the very end of the regular daily schedule. All involved personnel wore personal protective equipment (PPE), including dedicated garments, goggles, and NIOSH N95 respirators (FFP2 equivalent). A chest planning CT scan was performed, organs at risk (OARs) and whole-lung target volume were contoured. A 1-Gy prescription was opted for and planning with the VMAT technique was performed. Procedure duration included 10 min of patient positioning, 1:20 min CBCT imaging verification, and 1:30 min beam-on time. Taking the same previously described measures, the patient was transported to the intensive care unit (ICU) for continuing medical management. Quaternary ammonium was employed for disinfection and ultraviolet light was additionally used to cover the simulation CT and treatment bunkers.

The patient was kept under observation due to a potential need of intubation and assisted ventilation. Oxygen therapy was continued with a high-flow nasal cannula (HFNC) at 50 L/min due to persistent altered ABG values. Medical therapy was intensified with hydroxychloroquine 400 mg bid and azithromycin 500 mg bid; additionally, the enoxaparin dose was intensified (60 mg sc bid).

Three days after RT (sixth hospitalization day), the patient showed improvement in respiratory patterns and a persistent, although ameliorated, cough. HFNC management was de-escalated to a low-flow system with 30% FiO_2_. ABG parameters showed PaO_2_ 90.5 mm Hg and Pa/FiO_2_ 226.25. Seven days after treatment the patient was discharged from the ICU to continue management in a lower-complexity area. No RT-related toxicity was seen during the process.

**Fig. 1:**
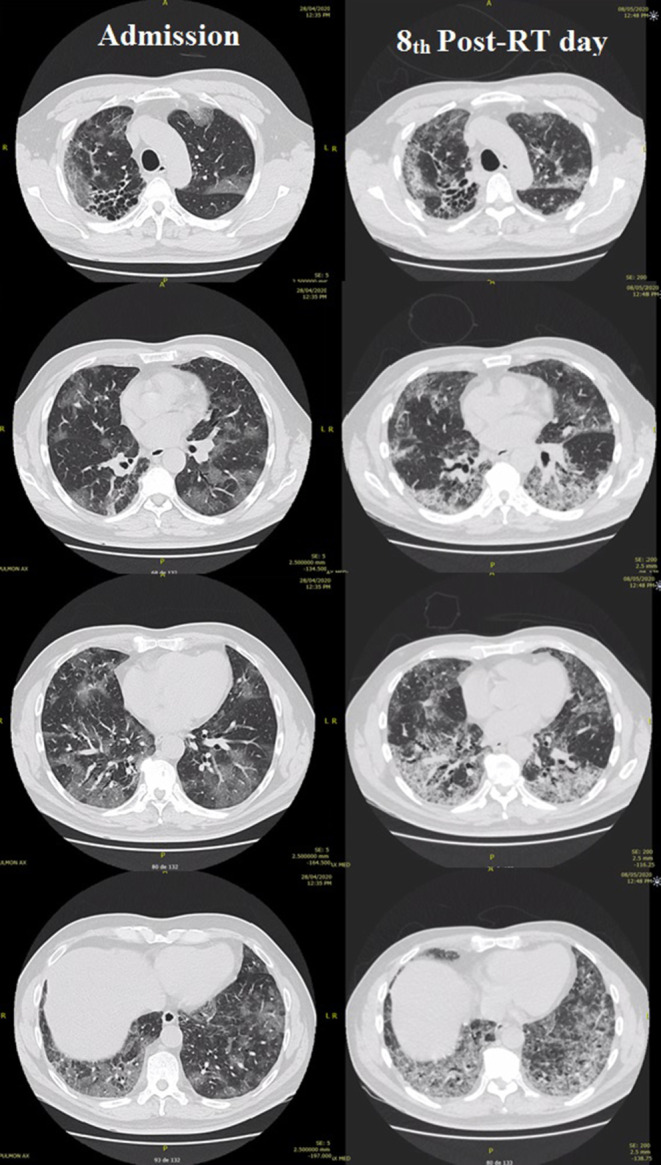
Baseline and eighth post-RT day imaging. Evident worsening of the inflammatory process can be observed; however, a dissociation between these finding and the good clinical evolution of the patient is to be noted

Imaging control did not show a clinical correlation: comparing the first CT at admission with the eighth post-RT day, the latter displayed a stronger interstitial inflammatory pattern (Fig. 1).

## Radiotherapy physics and planning considerations

**Fig. 2:**
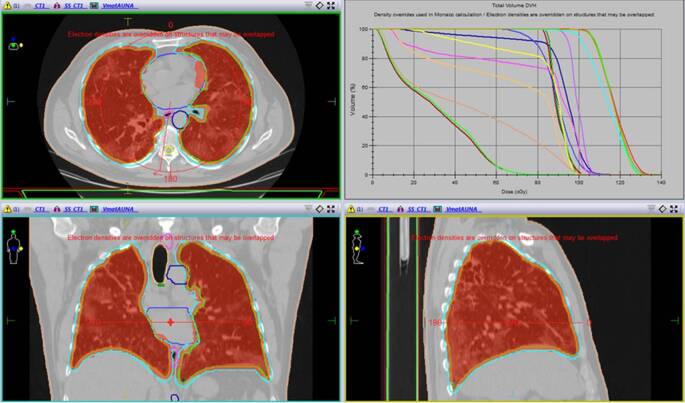
1‑Gy isodose volume distribution and dose–volume histogram showing a conformal and homogeneous distribution profile on the desired target volume

**Fig. 3:**
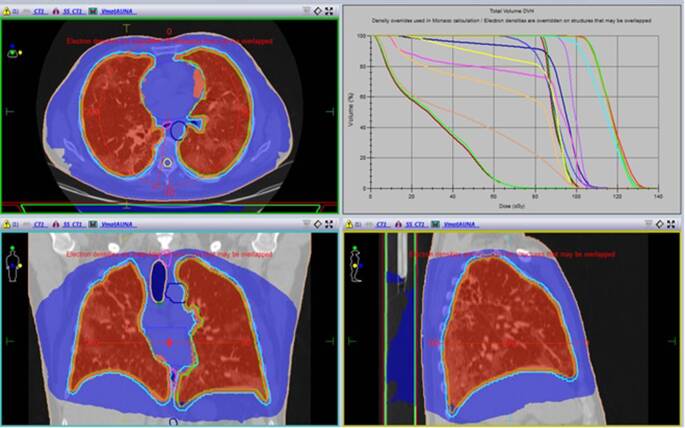
0.5 Gy isodose volume distribution and dose–volume histogram. The lower dose distribution profile reaches healthy surrounding structures without major clinical impact

A chest CT scan with 2.5 mm thick slices was acquired for planning purposes, accounting for a total set of 143 images. The hands-up position was determined and non-forced expiration was intended. Delineation of OARs and whole-lung CTV was performed on a contouring system (Monaco v.5.11.02, Elekta AG, Sweden). A circumferential 5‑mm and craniocaudal 10-mm PTV expansion was created. The contoured OARs included the heart, esophagus, spinal cord, and trachea. Additionally, the aorta, bronchi, and carina were considered for documentation motives (Table 2). A 1-Gy single dose was prescribed to the PTV (Fig. 2). Planning was performed with the Monte Carlo algorithm, for a 6-MV nominal energy delivered with a 360° arc and 90° collimator position (Elekta Infinity, Elekta AG) for better conformity achievement. Total estimated monitor units (MU) were 692.24. The approximated planning time was 30 min. Equivalent uniform dose (EUD) constraints were employed and priority for heart avoidance was given. Isodose volumes for 1 Gy and 0.5 Gy and dose–volume histograms (DVH) can be seen in Figs. 2 and 3. In addition, the target coverage and OAR dosimetry details can be seen in Table 3.

**Table 3:** Target coverage and OAR dosimetry

	Volume (cc)	Min. dose (cGy)	Max. dose (cGy)	Median dose (cGy)	Dose/volume (cGy)
CTV	4392.124	51.6	138.2	116.1	–
PTV	5582.966	36.5	138.2	113	D95–100
					D50–113.9
Heart	615.605	59.6	123.7	89.1	D2–105.6
Trachea	47.747	9	104.4	66.7	D2–98.2
Spinal cord	28.493	16.1	102.0	82.5	Dmax 100.7
Esophagus	28.195	7.9	109.8	78.2	D2–104
Carina	7.857	82.2	100.6	89.6	Dmax 98.4

*OAR* organ at risk, *CTV* clinical target volume, *PTV* planning target volume, *Dmax* maximum dose

## Literature review and discussion

This first reported case to our knowledge in our country and the region raises the question of the effectiveness of RT to treat COVID-19 pneumonia patients. Due to the lack of contemporary data, it is still difficult to define the safety profile and best treatment timing in terms of prompt or delayed management. Regarding toxicity, the expected secondary effects after whole-lung irradiation are well known by radiation oncologists according to the delivered dose [9, 10]. Doses lower than 1 Gy should not be of major concern for short- or long-term follow-up. Lessons obtained from preclinical and clinical studies, the former performed for the first time over 100 years ago, have already established the basis for this treatment modality [11]. Experiments in cats and mice performed in the 1940s showed a beneficial protective effect in animals exposed to 0.5–1 Gy 24 h after virus inoculation. On the contrary, no greater difference was observed with exposure after 48 h, suggesting a major improvement in cytokine release syndrome (CRS) regulation with early treatment onset [12, 13]. A more recent investigation assessed the impact of a 1-, 2‑, and 3‑Gy single fraction in a murine model, in order to simulate the impact of radiation exposure in astronauts. After 2‑year follow-up, neither of the selected doses caused caspase‑3 overexpression nor ceramide species accumulation, which directly induce pneumocyte apoptosis and subsequent fibrosis [14].

The radiobiological rationale for adopting RT as an approach to counteract CRS encompasses a variety of mechanisms. COVID-19 severe acute respiratory syndrome (SARS) pathogenesis is directly related to T helper 1 cell (Th1) activation, which subsequently initiate the dysregulated inflammatory cascade through different factors such as granulocyte colony-stimulating factor (G-CSF), IL‑6, IL‑7, IL-10, and tumor necrosis factor α (TNF-α) amongst others, and in addition to phagocytic activity, contribute to upregulating the cytokine storm [15–17]. An observed pattern in severe cases points to major damage to tissues expressing high concentrations of angiotensin-converting enzyme 2 (ACE2), mainly type II pneumocytes; therefore, the renin–angiotensin–aldosterone cascade is consequently added as an extra factor by further activating macrophages and granulocytes and promoting continuous interstitial inflammation [18, 19]. The anti-inflammatory properties of RT include polarization of macrophages to an M2 phenotype (leukocyte adhesion mediator), and downregulation of nitric oxide, TNF-, and TGF‑α. Besides, the upregulation of heme oxygenase, IL-10, TNF‑β, NFκβ, and apoptosis mechanisms, as well as T‑regulatory cell enhancement, have been postulated as co-adjuvant mediatory reactions [20–22]. Moreover, LD-RT might prevent accelerated viral drug-related mutation, which has been recently described due to increased selective pressure, while potentially improving the immune response by means of the previously described mechanisms and enhanced RNA damage compared to antiviral therapy [23, 24].

In the clinical setting, data about nonmalignant pathologies were published during the past century. The comprehensive review published by Calabrese et al. describes a series of publications regarding infectious diseases, including bacterial and interstitial pneumonia [6, 25]. An example is the study published by Oppenheimer in 1943. Satisfactory clinical outcomes were obtained after treating 56 patients with a 0.5-Gy equivalent, doses that were previously reported to satisfactorily treat bacterial lung conditions by Chamberlain [26]. Differences between three subgroups were reported, as patients who were treated from 2–5 days and 6–14 days had full symptom resolution. However, with treatment onset after 14 days, the therapy failed to completely resolve symptoms in 50% of the subjects. No fatalities were recorded [27].

It is also to be remarked that the overall COVID-19 intubation-related mortality rate varies between 30 and 70%, according to specific geographical regions [5, 15, 16]. No major data are available at the moment in terms of lung injury secondary to mechanical ventilation, although this still carries inherent risks [28, 29]. Preventive therapeutic approaches to avoid intubation seem of relevance, as this is associated with elevated mortality per se. However, this premise should not be taken lightly, due to the delicate balance between noninvasive and invasive ventilatory management in these terms. It has been previously demonstrated that noninvasive ventilation and delayed intubation, when indicated, could also yield lethal consequences in SARS management [30, 31].

Regarding irradiation technique selection, a three-arc VMAT plan was preferred over APPA and IMRT-based fields, in order to guarantee whole-lung tissue coverage and OAR (mostly cardiovascular structures) sparing, while simultaneously maintaining the beam-on time as short as possible [32–34]. It should also be noted that positioning and verification times must be accounted for; therefore, expected target coverage, OAR exposure, and image verification technique are factors to be included in decision-making. Additional criteria according to available technology and logistic features could be considered. Furthermore, it is important to highlight that the exposure threshold remains controversial for translation into clinical effects. Previous differing publications have described either none or developing of detrimental effects after delivering a 0.5 Gy mean dose to the heart [35, 36]. Additionally, the risk of developing secondary cancers after LD-RT from previously reported benign entity treatments has been estimated to be below 1% [37]. Weighing up all these considerations and due to the rapid deterioration of these patients’ status, prioritizing the management of the acute event, in order to potentially save the patient’s live, is of major relevance.

The compassionate treatment offered to the patient and the possible related response in terms of avoiding invasive maneuvers showed clinical improvement from day 2 after RT. This is also correlated with the observed continuous decrease in inflammatory markers. The initial elevation of serum markers has been described previously, potentially establishing the first downregulator signal and starting point for further inflammatory control; however, this should be further elucidated [38]. Similarly, the observed mild lymphopenia could be derived from the same phenomenon, although the development of COVID-19 has also been reported as a direct cause of this alteration, as for other viral infections [39]. Despite this, the case’s evolution time would indirectly point to a positive effect of RT, due to the usual status worsening of patients after the seventh day. However, no conclusions but only hypothesis-generating premises could be drawn. 

Close follow-up, ICU management including continuous ABG, and imaging control are mandatory. The intervention decision should be taken on a multidisciplinary basis and with express consent of the patients, balancing potential benefits and risks, preferably encompassed by a prospective clinical protocol. Ethical concerns are raised as well, due to the rapid deterioration of patients’ status and the need for early decision-making. In desperate need of positive outcomes, errors from selecting inadequate treatment should not be repeated. Recently published evidence from different groups has demonstrated not only the lack of effectiveness but also the risks of employing hydroxychloroquine and azithromycin [40–44].

Despite the previously presented evidence and pathophysiological plausibility, and on the basis of this novel situation, no agent-specific clinical data addressing the possible long-term secondary effects of treating this particular group of patients are available, nor is it known if a synergy between the COVID-19-induced CRS and RT could impact the incidence of long-term secondary events. This may be taken into consideration when prescribing RT in this setting.

The importance and responsibility of establishing properly designed study protocols to clarify this hypothesis falls to the multidisciplinary team and joint efforts from leading scientific societies. Consensus for patient management and health personnel protection is urgently needed. Results from ongoing trials (NCT04377477, NCT04380818) will elucidate the effectiveness of this therapeutic tool regarding improvement in Pa/FiO_2_ parameters, hospital stay, and ICU admission rates. Assessing rates of invasive management requirements after RT as a specific endpoint is also recommended.

## Conclusion

Radiotherapy arises as a promising option for COVID-19 pneumonia management. Prospective data from a larger cohort of patients are needed to confirm the safety profile and effectiveness of this approach in this specific group of patients.
